# Putative *cis*-regulatory elements in genes highly expressed in rice sperm cells

**DOI:** 10.1186/1756-0500-4-319

**Published:** 2011-09-05

**Authors:** Niharika Sharma, Scott D Russell, Prem L Bhalla, Mohan B Singh

**Affiliations:** 1 Plant Molecular Biology and Biotechnology Laboratory, Australian Research Council Centre of Excellence for Integrative Legume Research, Melbourne School of Land and Environment, University of Melbourne, Parkville, Victoria 3010, Australia; 2 Department of Botany and Microbiology, University of Oklahoma, Norman, OK 73019, USA

**Keywords:** *cis*-regulatory elements, plant reproduction, male gamete, gene expression, *Oryza sativa*

## Abstract

**Background:**

The male germ line in flowering plants is initiated within developing pollen grains via asymmetric division. The smaller cell then becomes totally encased within a much larger vegetative cell, forming a unique "cell within a cell structure". The generative cell subsequently divides to give rise to two non-motile diminutive sperm cells, which take part in double fertilization and lead to the seed set. Sperm cells are difficult to investigate because of their presence within the confines of the larger vegetative cell. However, recently developed techniques for the isolation of rice sperm cells and the fully annotated rice genome sequence have allowed for the characterization of the transcriptional repertoire of sperm cells. Microarray gene expression data has identified a subset of rice genes that show unique or highly preferential expression in sperm cells. This information has led to the identification of *cis*-regulatory elements (CREs), which are conserved in sperm-expressed genes and are putatively associated with the control of cell-specific expression.

**Findings:**

We aimed to identify the CREs associated with rice sperm cell-specific gene expression data using *in silico *prediction tools. We analyzed 1-kb upstream regions of the top 40 sperm cell co-expressed genes for over-represented conserved and novel motifs. Analysis of upstream regions with the SIGNALSCAN program with the PLACE database, MEME and the Mclip tool helped to find combinatorial sets of known transcriptional factor-binding sites along with two novel motifs putatively associated with the co-expression of sperm cell-specific genes.

**Conclusions:**

Our data shows the occurrence of novel motifs, which are putative CREs and are likely targets of transcriptional factors regulating sperm cell gene expression. These motifs can be used to design the experimental verification of regulatory elements and the identification of transcriptional factors that regulate sperm cell-specific gene expression.

## Introduction

As in animals, flowering plant sperm cells are small cells that fuse with the egg during fertilization. The sperm cells produced within developing pollen remain enveloped by much larger vegetative cell. Typically, sperm cells occupy < 0.1% of the pollen grain volume. The germination of pollen leads to the extension of the vegetative cell wall to produce a pollen tube, which grows via tip elongation to deliver sperm cells to the embryo sac. Until recently, the condensed appearance of chromatin associated with its small cytoplasmic volume was considered to reflect transcriptional quiescence of sperm cells. Recent developments in techniques to isolate sperm cells from pollen [1] along with the availability of high-throughput genomic and transcriptomic tools have allowed for the analysis of gene expression in these small cells. The latest reports of gene expression studies in Arabidopsis [2], maize [3], *Plumbago *and lily [4] sperm cells have shown that the initial views regarding sperm cells as transcriptionally quiescent were not correct [5]. Recent investigations have revealed that sperm cells have highly distinct expression profiles from vegetative stages. Our recent microarray investigations of sperm cells in *Oryza sativa *exposed distinct expression profiles for many genes in male germ line cells, including *GEX1*, *GEX2*, and *GCS1/HAP2*, a set of genes whose sperm specificity is conserved in Arabidopsis, lily, and rice. These expression studies exhibit the conserved and sophisticated control of molecular mechanisms in sperm cell development.

Cellular signaling pathways often consist of interacting loops of transcription factors and *cis*-regulatory DNA elements that direct the expression of target genes. We aimed to take a computational approach to investigate candidate *cis*-regulatory elements (CREs) in a cluster of co-expressing genes in rice sperm cells. In this paper, we discuss the functions of these CREs in plant biology, including the regulation of cell cycle and reproductive development. This study will accelerate the functional characterization of CREs and their interacting transcription factors and will serve as a step forward in exploring systems biology networks in transcriptional regulation.

Transcriptional regulation of organ-specific and cell-specific gene expression is mediated by the recruitment of transcription factors to CREs. Transcription factors interact with specific DNA elements, other factors and the basal transcriptional machinery to regulate the expression of target genes. In plants, transcriptional regulation is mediated by more than 1500 transcriptional factors; each of these factors controls the expression of tens or even thousands of target genes in complex signaling networks [6]. Along with transcription factors, CREs are functional DNA motifs or elements that establish conspicuous temporal and spatial transcriptional activity. Identifying and understanding the functions of such CREs are essential for elucidating the mechanism by which cells perceive and correctly respond to their environment and participate in organism growth and development.

Microarray gene expression data can help to identify groups of co-expressed genes. Clusters of such co-expressed genes are assumed to be co-regulated and upstream sequences of these genes are likely to share common DNA motifs. Presumed upstream regulatory regions of arbitrary length can be used to identify candidate DNA motifs. Because of their importance, we studied multiple motifs over-represented in the promoters of a co-expressed gene cluster. This type of investigation can illustrate the utility of the co-expression-driven prediction of CREs as a means to begin deciphering transcriptional networks. A number of algorithms and bioinformatics tools have been developed to identify potential CREs in the regulatory sequences of co-expressed genes. Most computational approaches assume that co-regulated genes should contain similar CREs in their upstream regulatory regions at statistically significant levels. The transcriptional control of gene expression depends on a balance between activating and repressing regulatory components in upstream regulatory regions. Hence, CREs play a central role in regulating gene expression by integrating signals at the DNA-level upstream of a target gene. Our study presents an *in silico *analysis of 1-kb upstream promoter sequences that regulate sperm cell-expressing genes in rice.

## Materials and methods

One Kb upstream DNA sequences of top 40 genes that showed high expression in sperm cells of rice http://www.ricechip.org were mined from TIGR (The Institute of Genome Research) database release 6.1 ftp site ftp://ftp.plantbiology.msu.edu/pub/data/Eukaryotic_Projects/o_sativa/annotation_dbs/pseudomolecules/version_6.1/all.dir/all.1kUpstream.

Analysis of one Kb upstream promoter sequences for locating known CREs was conducted using SIGNALSCAN program available in Plant *cis*-Regulatory DNA Elements (PLACE), and database of *cis*-regulatory element motif http://www.dna.affrc.go.jp/PLACE/[7,8]. The database contains mainly plant motifs extracted from the published reports in the literature.

CREs for more extensive analysis were selected based on the frequency of their occurrence. These results were further refined to find CREs present in 80% of the gene dataset. A comprehensive map depicting precise locations of these abundantly occurring CREs was also prepared by placing them on scales of upstream promoter sequences manually.

Unknown novel CREs in the sequence dataset were also detected using MEME (Multiple EM for Motif Elicitation) tool version 4.4.0 http://meme.sdsc.edu/meme/intro.html MEME analyses sequences for similarities and produces a description for each pattern or motif it discovers [9]. A motif is a sequence pattern that occurs repeatedly in a group of related protein or DNA sequences. MEME represents motifs as position-dependent letter-probability matrices that describe the probability of each possible letter at each position in the pattern. Individual MEME motifs do not contain gaps.

To complement searches for novel CREs detection, we also used the Mclip program developed by Frickey and Weiller (2007), which we operated online at: http://bioinfoserver.rsbs.anu.edu.au/utils/mclip/ modifying parameters by using E-values of 1e-13 as cut-off values for motif matching and motif alignment (rather than the default 1e-3). End gaps were allowed to permit longer motifs to be considered. For validation, we selected sperm cell probe sets reported as three unanimous presence calls ("PPP") in sperm microarray results, accompanied by corresponding unanimous absence calls ("AAA") in pollen (vegetative) cells and seedlings. The selected sperm probe sets were matched to loci. Loci annotated as containing transposable element sequences were omitted as a precaution to reduce the likelihood of finding sequences that may be disproportionately be amplified owing to transposable element activity. For the remaining loci after screening, 1 K upstream sequences were obtained as FASTAs. We considered the occurrence of all possible 8-nt motifs by constructing a matrix of all finds. To the extent that a "found" motif can be considered as a discrete event, the Poisson distribution is appropriate to determine if expression is distorted from that occurring by chance and thus it is possible to assign a calculated p value. The motifs that were found through Mclip were then screened against known CREs, and only novel sequences were considered.

## Results and Discussion

The investigation and identification of *cis*-regulatory elements (CREs) in the promoters of high expressing sperm cell genes will aid in deciphering the function of these genes in male gametophyte and the sperm cell development in rice. In addition, the results can be used for further analysis of gene networks in male gametophyte development in plants.

### Highly expressed transcripts in rice sperm cells

Some initial reports have described differentially expressed genes in pollens, anthers and male gamete cells in plants [10]. However, deciphering gene expression in plant sperm cells was not possible due to the unavailability of protocols allowing for their meaningful characterization. With the development of techniques to collect sperm cells and compare gene expression profiles between pollens, anthers, sperm cells and sporophytic tissues, sperm cells are now known to have distinct expression profiles. The sperm cell-expressed transcripts are functionally categorized for their involvement in DNA repair, cell cycle progression and ubiquitin-mediated proteolysis.

Conserved genes with especially enriched sperm cell expression in flowering plants include Arabidopsis GEX-1 (gamete expressed protein 1), which encodes a transmembrane domain-containing protein, and GEX-2 (gamete expressed protein 2) [5]. GCS-1 (generative cell specific 1), also known as HAP2, is expressed only in haploid sperms, contributes to pollen tube guidance and is required for fertilization [11].

We considered a dataset of CREs consisting of 1-kb upstream promoter sequences of 40 sperm cell-expressed genes in rice, which are located at different positions on 12 chromosomes. Information about the genes and their positions, descriptions and expression values are presented in Table 1. These genes are highly expressed in rice sperm cells as detected by microarrays with intensity values http://www.ricechip.org.

**Table 1:** List of highly expressing sperm cell genes in rice.

S. No	Probe Set ID	Gene ID	Location 5'-3' (Base pair)	Nucleotide Length (Base Pair)	Gene Product Description	Intensity log_2_
1	Os.10737.1.S1_at	LOC_Os05g18730.1	10854579 - 10847943	2169	generative cell specific-1, putative, expressed	15.4436

2	Os.54874.1.S1_at	LOC_Os09g27040.1	16444436 - 16446851	1920	GEX1, putative, expressed	12.808

3	OsAffx.17894.1.S1_at	LOC_Os09g25650.1	15414547 - 15407935	3291	GEX2, putative, expressed	14.6951

4	Os.41333.1.A1_at	LOC_Os01g42060.1	23845997 - 23849282	1908	expressed protein	12.4728

5	Os.53049.1.S1_at	LOC_Os04g46490.1	27391780 - 27390795	810	aquaporin protein, putative, expressed	15.7476

6	Os.26448.1.A1_at	LOC_Os03g08070.1	4125935 - 4119369	2658	copper-transporting ATPase PAA1, putative, expressed	11.0327

7	Os.21018.1.S1_at	LOC_Os09g35720.1	20547711 - 20541270	2397	generative cell specific-1, putative	14.8986

8	OsAffx.26224.1.S1_s_at	LOC_Os04g29090.1	17091173 - 17086877	1989	FAD-binding and arabino-lactone oxidase domains containing protein, putative, expressed	12.3096

9	Os.18560.1.S1_at	LOC_Os05g01500.1	291425 - 296877	1641	tubulin-specific chaperone E, putative, expressed	11.2132

10	OsAffx.3617.1.S1_at	LOC_Os03g55890.1	31822145 - 31818577	1950	ternary complex factor MIP1, putative, expressed	10.1043

11	OsAffx.2553.1.S1_at	LOC_Os02g09580.1	4931298 - 4935969	1308	OsFBX39 - F-box domain containing protein	9.7882

12	Os.51974.1.S1_at	LOC_Os06g20860.1	12052626 - 12049870	2580	paramyosin, putative, expressed	11.6793

13	Os.50267.1.S1_at	LOC_Os08g34640.1	21757895 - 21754127	3339	receptor-like protein kinase precursor, putative, expressed	12.8324

14	Os.55267.1.S1_at	LOC_Os03g44630.1	25129797 - 25129252	429	plastocyanin-like domain containing protein, putative, expressed	13.8559

15	Os.9559.1.S1_at	LOC_Os03g37570.1	20838322 - 20836787	633	expressed protein	13.1792

16	OsAffx.2680.1.S1_at	LOC_Os02g19180.1	11184386 - 11182943	915	ZOS2-06 - C2H2 zinc finger protein	13.3352

17	Os.52171.1.S1_at	LOC_Os06g38950.1	23111994 - 23104560	2850	ABC transporter, ATP-binding protein, putative, expressed	15.1547

18	Os.32737.1.S1_at	LOC_Os11g08440.1	4439113 - 4441939	1734	DnaK family protein, putative, expressed	12.267

19	Os.38984.1.S1_at	LOC_Os01g23580.1	13236319 - 13244856	2322	inorganic H+ pyrophosphatase, putative, expressed	12.9446

20	Os.23286.1.A1_at	LOC_Os10g02920.1	1190765 - 1188954	678	cytochrome b561, putative, expressed	12.3191

21	OsAffx.26489.1.S1_at	LOC_Os04g46760.1	27543355 - 27541189	1050	trehalose phosphatase, putative	12.2378

22	Os.53437.1.S1_at	LOC_Os03g45980.1	25991611 - 25992474	864	expressed protein	12.459

23	Os.46544.1.A1_at	LOC_Os10g25060.1	12830509 - 12828658	588	expressed protein	13.4751

24	Os.9431.1.A1_a_at	LOC_Os08g16610.1	10159371 - 10164170	2187	Rad21/Rec8 like protein, putative, expressed	15.1214

25	OsAffx.12136.1.S1_at	LOC_Os02g20530.1	12106516 - 12110223	1812	hypothetical protein	12.4087

26	OsAffx.24724.1.S1_x_at	LOC_Os02g44599.1	27021672 - 27021157	516	expressed protein	12.4125

27	Os.4125.1.S1_at	LOC_Os12g38460.1	23577850 - 23572909	3399	RNA recognition motif family protein	12.8627

28	Os.54137.1.S1_at	LOC_Os02g08080.1	4268788 - 4264756	1428	expressed protein	12.6933

29	OsAffx.31616.1.S1_at	LOC_Os12g06480.1	3123385 - 3117268	2376	PHD-finger family protein, expressed	13.5878

30	Os.52287.1.S1_at	LOC_Os02g02800.1	1067067 - 1068845	762	AGAP001222-PA, putative, expressed	12.4595

31	OsAffx.14496.1.S1_at	LOC_Os05g02030.1	590266 - 591787	582	OB-fold nucleic acid binding domain containing protein, putative	11.1188

32	Os.50552.1.S1_at	LOC_Os08g35700.1	22526032 - 22519854	2706	Leucine Rich Repeat family protein, expressed	13.9674

33	OsAffx.28262.1.S1_at	LOC_Os07g04520.1	1993662 - 1991627	1554	protein kinase, putative, expressed	12.6217

34	Os.56612.1.A1_x_at	LOC_Os05g11980.1	6852880 - 6843407	3624	timeless protein, expressed	11.097

35	Os.34965.2.S1_s_at	LOC_Os06g07130.1	3407672 - 3417233	2043	SHR5-receptor-like kinase, putative, expressed	10.7422

36	Os.54810.1.A1_at	LOC_Os08g28080.1	17129894 - 17119408	2514	coatomer subunit delta, putative, expressed	12.4403

37	Os.52821.1.S1_at	LOC_Os11g37200.1	21516300 - 21519963	777	transmembrane BAX inhibitor motif-containing protein, putative, expressed	14.25

38	Os.54486.1.S1_at	LOC_Os05g03320.1	1352666 - 1348957	1686	expressed protein	12.624

39	Os.10491.1.S1_at	LOC_Os03g04690.1	2227622 - 2226738	885	expressed protein	10.5236

40	Os.23535.1.A1_at	LOC_Os08g05820.1	3124469 - 3119480	1803	monocopper oxidase, putative, expressed	15.4961

### Identification of Known CREs

CREs were extracted from the input dataset based on previously published reports and databases of regulatory elements and motifs. The PLACE database for *cis*-acting regulatory DNA elements and the SIGNALSCAN search tool were used to estimate the mode of gene regulation and to find the regulatory and other pertinent regions in regulatory promoter sequences in genes highly expressed in rice sperm cells.

### Abundant CREs

**Figure 1:**
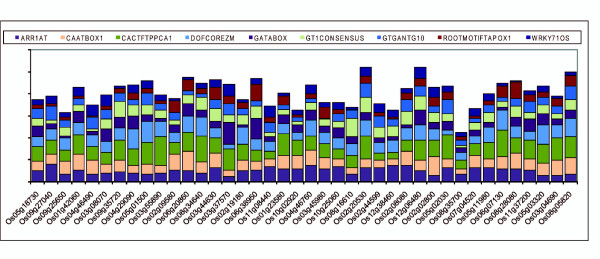
**Duplication frequency of 9 most abundant CREs in top 40 highly expressed genes in sperm cell**.

Evaluation of 1-kb upstream promoter regions (-1 to -1000) of rice sperm cell-expressing genes using SIGNALSCAN resulted in the identification of 223 types of CREs. Nine of the CREs were found in all of the 40 genes: ARR1AT (5'-NGATT-3'), CAATBOX1 (5'-CAAT-3'), CACTFTPPCA1 (5'-CACGTG-3'), DOFCOREZM (5'-AAAG-3'), GATABOX (5'-GATA-3'), GT1CONSENSUS (5'-GRWAAW-3'), GTGANTG10 (5'-GTGA-3'), ROOTMOTIFTAPOX1 (5'-ATATT-3') and WRKY71OS (5'-TGAC-3'). The duplication frequency of these CREs in all 40 genes is depicted in Figure 1 and the numbers are given in Additional file 1. Among these nine CREs, CACTFTPPCA1 is the most abundant CRE, with duplications in the range of 2 to 26 in the 1-kb upstream region of each highly up-regulated sperm cell promoter, followed by DOFCOREZM, ARR1AT and CAATBOX1 with 595, 460, 417 and 416 duplications, respectively, in all 40 sequences.

Recently, it has been experimentally explained that *AGO5 *promoter drives gene specifically expressed in Arabidopsis sperm cells [12] during gametophyte development. All 9 most abundant CREs discussed above were also found in Arabidopsis *AGO5 *promoter 1 Kb upstream region when analyzed with SIGNALSCAN function of PLACE. For instance there are 6 ARR1AT, 12 CACTFTPPCA1, 12 GT1CONSENSUS and 22 DOFCOREZM.

CACTFTPPCA1, the most duplicated CRE, is a tetranucleotide motif responsible for mesophyll-specific gene expression of C4 phosphoenolpyruvate carboxylase gene in C4 plants [13]. It is a key component of *Mem1 *(mesophyll-expression module 1) in *Flaveria trinervia *but might have a different role in C3 plants, such as rice.

DOFCOREZM is the target binding site of Dof proteins, which are specific DNA-binding proteins associated with the expression of multiple genes in plants [14]. Dof proteins also differentially regulate diverse promoters in a variety of plant tissues [15]. Binding sites for Dof transcription factors have been recorded in the upstream sequences of *GEX1 *and *GEX2*, two genes showing sperm-specific expression in Arabidopsis [5]. Interestingly, rice homologues of both of these genes are represented among the 40 highly expressed genes of rice sperm cells.

ARR1AT (ARR1-binding element) is found in both Arabidopsis and rice. ARR1 and ARR2 are cytokinin response regulators that function as transcriptional activators [16]. AGATT has also been reported to be in the promoter of the rice non-symbiotic haemoglobin-2 (*NSHB*) gene [17]. CAATBOX1, the CAAT promoter consensus sequence is responsible for the tissue-specific promoter activity of the pea legumin gene *LegA *[18].

GATABOX, which are GATA motifs, are known to be required for high level, light regulated and tissue specific gene expression. GATA transcription factors are a group of DNA-binding proteins distinguished by a zinc finger motif, which have been implicated in light and nitrate-dependent transcription control [19]. The zinc finger transcription factor genes are among the genes showing the highest expression level in *Arabidopsis *sperm cells [2]. However, the G-Box and GATA elements are reported to occur several times on average in every potential upstream regulatory region [20]. GATA transcription factors are reported to bind the CaMV 35S promoter and are conserved in cab promoters as well [21].

GT1CONSENSUS recognizes GT-1 proteins, which have tri-helix DNA-binding domains, are conserved in plant nuclear genes and have diverse functions [22,23]. GT elements are ubiquitously expressed and show complex regulatory features of plant gene transcription [24]. GTGANTG10, is a GTGA motif found in the promoter of tobacco late pollen gene *g10*. The tobacco gene *g10 *is preferentially and maximally expressed in mature pollen, shows homology to pectate lyases, and is the putative homologue of the tomato gene *lat56 *[25]. ROOTMOTIFTAPOX1 is a motif found in the rolD promoter of *Agrobacterium rhizogenes*. The *rolD-gus *genes were found to have a distinctive expression pattern in roots [26].

WRKY71OS is a binding site of rice *WRKY71*, a transcriptional repressor of the gibberellin signaling pathway [27]. It is a core of TGAC-containing W-box of the *Amy32b *promoter within *PR-10 *genes [28]. Because *WRKY *ESTs are highly abundant in plant cDNA libraries generated from floral and embryonic material, WRKY transcription factors are presumed to have vital functions in these tissues [29]. Family members of WRKY transcription factors appear to be involved in the regulation of various physiological programs that are unique to plants, including pathogen defense, senescence, trichome development plant growth and development. The rice WRKY gene superfamily has also been implicated in the regulation of abscisic acid signaling in aleurone cells [30].

**Figure 2:**
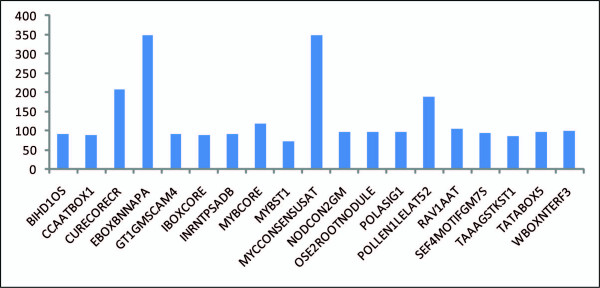
**Duplication numbers of other abundant 19 CREs in 80% of top 40 highly expressed genes in sperm cell**.

The other 19 CREs present in almost 80% of genes and abundant in distribution were BIHD1OS (5'-TGTCA-3'), CCAATBOX1 (5'-CCAAT-3'), CURECORECR (5'-GTAC-3'), EBOXBNNAPA (5'-CANNTG-3'), GT1GMSCAM4 (5'-GAAAAA-3'), IBOXCORE (5'-GATAA-3'), INRNTPSADB (5'-YTCANTYY-3'), MYBCORE (5'-CNGTTR-3'), MYBST1 (5'-GGATA-3'), MYCCONSENSUSAT (5'-CANNTG-3'), NODCON2GM (5'-CTCTT-3'), OSE2ROOTNODULE (5'-CTCTT-3'), POLASIG1 (5'-AATAAA-3'), POLLEN1LELAT52 (5'-AGAAA-3'), RAV1AAT (5'-CAACA-3'), SEF4MOTIFGM7S (5'-RTTTTTR-3'), TAAAGSTKST1 (5'-TAAAG-3'), TATABOX5 (5'-TTATTT-3') and WBOXNTERF3 (5'-TGACY-3'). Duplication numbers of these CREs are delineated in Figure 2, and the values are given in Additional file 2. Taken together, these CREs represent some of the major categories abundantly distributed and duplicated in the 1-kb upstream regulatory sequences of sperm cell-specific genes in *O. sativa*. The most over-represented CREs were EBOXBNNAPA, MYCCONSENSUSAT, CURECORECR, POLLEN1LELAT52 and MYBCORE. All these 19 CREs were also found to be present in *AGO5 *promoter sequence, which induces sperm cell specific expression in Arabidopsis [12].

EBOXBNNAPA is an E-box sequence, which has been reported in *Brassica napus *[31]. Also known as an RRE element, this CRE is responsible for light responsiveness and is regulated by bHLH and the MYB-transcription factor in directing tissue-specific expression [32]. MYCCONSENSUSAT is a MYC recognition site found in the promoters of the dehydration-responsive gene *rd22 *and many other genes in Arabidopsis [33]. This CRE also regulates the transcription of Arabidopsis genes under cold conditions by a MYC-like bHLH transcriptional activator [34]. Few bHLH transcription factors have been detected in *Arabidopsis *sperm cells and none have been found in pollen, suggesting that they might play a specific role in male gamete formation [2]. CURECORECR (GTAC) is located in the core of a CuRE (copper-response element), which is found in the *Cyc6 *and *Cpx1 *genes in *Chlamydomonas*. This CRE is also involved in oxygen-response of these genes [35]. POLLEN1LELAT52 is a regulatory element responsible for pollen-specific activation of the tomato *lat52 *gene. This CRE has also been found in the promoter of tomato endo-beta-mannanase gene during late stages of anther development [36]. MYBCORE is a binding site for two plant MYB proteins, AtMYB1 and AtMYB2, which were isolated from Arabidopsis. AtMYB2 is involved in the regulation of genes responsive to water stress [37]. MYB-type transcription factors are reported to have relatively high expression in sperm cells [2]. Furthermore, MYB-proteins play crucial roles in cell proliferation and differentiation. Moreover, the MYB DNA-binding domains are relatively similar to those of transcription factors containing zinc finger, basic region/leucine zipper and basic region/helix-loop-helix domains [38].

BIHD1OS is a binding site of OsBIHD1, which is rice BELL homeodomain transcription factor [39] present in the nucleus, whose induction is associated with the resistance response in rice. Homeodomain transcription factors in Arabidopsis have also shown substantial expression in sperm cells [2]. CCAATBOX1 (CCAAT Box) and CONSTANS form a binding complex, which contains a functionally important domain and regulates flowering in *Arabidopsis *[40]. The CCAAT box is also found in the promoters of heat shock protein genes [41]. RAV1AAT is a consensus sequence of the Arabidopsis transcription factor target domain [42]. The RAV1 protein contains Ap2-like and B3-like domains [43]. AP2 is a member of the transcription factor family unique to plants and is a key regulator of several developmental processes in the plant life cycle including floral organ identity determination [44].

### Positionally biased CREs

Comprehensive mapping for the above 28 CREs and their locations in the 1-kb upstream region of each sperm cell specific gene regulatory sequence is shown in Additional file 3. The CREs seem randomly dispersed in the promoters with no particular pattern detected in their occurrence with respect to positions on 1-kb upstream sequences. But an interesting speculation is for some of the entries; comparatively large numbers of CREs are located on antisense strand as compared to sense strand. This observation is held true for Os05g18730.1, Os09g27040.1, Os11g08440.1, Os01g23580.1, Os05g11980.1 and Os08g08080.1.

### Low abundant CREs

In addition, 35 other unique CREs were only found in one of the 40 sperm cell-specific genes with just one or two duplications. These 35 CREs are presented in Additional file 4. Twenty-one genes contain these 35 unique CREs. In contrast to the 19 CREs found in 80% of sperm specific genes, 44 CREs are represented in about 5-10% of sperm-specific genes. These CREs are listed in Additional file 5.

### Identification of unknown CREs using MEME

**Figure 3:**
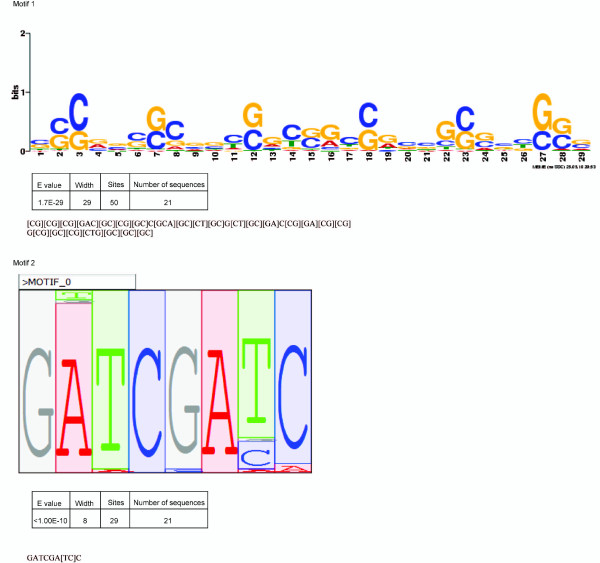
**Highest scoring novel motifs found by MEME and Mclip**.

Tools can be used for detecting unknown motifs in related DNA sequences. For example, MEME uses statistical modeling techniques to choose the width, number of occurrences and descriptions for each motif. Using MEME, the same dataset of high sperm cell-expressing genes produced one novel motif. The motif is found to present in 21 genes. These results are illustrated in Figure 3. Motif 1, which is present in 21 of the 40 genes, illustrates CG enrichment similar to that found in regulatory elements related to DNA methylation, which may be copied during replication. CpG islands are a common motif by which sequences are normally repressed. The prevalence of this motif suggests that sperm-restricted functions may function in repression within somatic tissue.

Some reports about CRE and promoter duplications indicate that the duplication numbers of CREs might participate in leveling the mRNA concentration or gene expression [44]. Thus, these investigations may reveal some clues about synchronous expression of this subset of genes in sperm cells.

*Cis*-regulatory elements of genes are closely related to spatiotemporal gene expression. CREs have been reported to control tissue-specific gene expression and condition-dependent gene expression similar to how heat shock elements (HSEs) control heat shock induction of gene expression [45].

Duplication and distribution of CREs might play a role in up- or down-regulation of specific genes. This data may be useful for understanding various phenomena in sperm cell development and will shed light on sperm cell development and expression. This study may facilitate our understanding of the molecular aspects of male gamete differentiation and function in flowering plants, such as rice.

### Search for novel CREs using Mclip

To complement searches for unknown transcription factor *cis*-regulatory sequences, we also conducted searches of the same 40 highly transcribed sperm loci using Mclip [46]. One-kb upstream sequences of these loci were used as seeds for the discovery of unknown conserved motifs, which were then screened against known *cis*-regulatory elements. Our results are illustrated in Figure 3. Interestingly, the program identified an expressed AT-rich sequence that matched a promoter sequence isolated from the upstream region of the *Plumbago *gene PzIPT [10]. The canonical TATA box was also represented in this sequence, but the deletion of the matched AT-rich *cis*-regulatory region resulted in a loss of expression. AT-rich motifs have also been reported in upstream regions of *GEX1 *and *GEX2 *[5] and were over-represented in 1-kb upstream sequences of sperm-expressed genes.

Novel motifs in the form of GATCGATC were present in 15 genes with 54 duplications, of which TCGA was most highly conserved. Similar sequences (ATCGATCG, TCGATCGA and CGATCGTA) were also represented frequently as 8-nt motifs and far exceeded random 8-nt motifs (ranging from 4.89E-21 to 3.3E-28).

In summary, we have revealed sequences (CREs) that may be responsible for driving high expression in male germ line cells. Though these sequence patterns require experimental validation (e.g. insertion deletion experiments), nevertheless, our current findings may open new avenues for studying the regulation of gene expression in male gametes of flowering plants.

## Competing interests

The authors declare that they have no competing interests.

## Authors' contributions

Conceived and designed the study: MS PB NS. Performed computational analysis: NS SDR.

Wrote the paper: NS PB SDR MS. All authors have read and approved the manuscript.

## Supplementary Material

Additional file 1 **Duplication numbers of 9 most abundant CREs in top 40 highly expressed genes in sperm cells of rice**. Based on the results of PLACE database SIGNALSCAN searches for total of 223 CREs, 9 of them are found to present in 1 Kb upstream regions of all 40 genes. The duplication numbers of those CREs are represented in this table. Frequency graph is also plotted for this distribution as shown in Figure 1. Click here for file

Additional file 2 **Duplication numbers of other 19 abundant CREs present in 80% of sperm cell expressing genes of rice**. Results of SIGNALSCAN searches gave 19 other CREs abundantly present in the dataset. The duplication numbers of these CREs are represented in this table. Frequency graph is also plotted for this distribution as shown in Figure 2. Click here for file

Additional file 3 **A map of 28 abundant CREs and their positions within 1 Kb upstream sequences**. SIGNALSCAN program of PLACE database identified the positions of CREs in the upstream regions of top 40 highly expressed genes in sperm cell of rice. These selected CREs were subjected to further extensive analysis for their duplication numbers and distribution across the upstream regions. The figure shows exact location of CREs present in 80% of the gene dataset. The blue bars above the horizontal black line indicate CREs on sense strand and the blue bars below the black line designates CREs on anti-sense strand. Click here for file

Additional file 4 **Unique CREs**. The analysis exhibited some unique CREs present in only one of the 40 sperm cell expressing genes with one or two duplications. These CREs were found in these specific sperm cell expressing genes. Click here for file

Additional file 5 **Peculiar CREs**. Besides abundant CREs present in 80% of the gene dataset, there are few others present in just 5-10% of rice sperm cell expressing genes. Click here for file
