# Supplementary material for: Putative cis-regulatory elements in genes highly expressed in rice sperm cells
Source: BMC Res Notes. 2011 Sep 5;4:319. doi: 10.1186/1756-0500-4-319 (PMC3224587; doi:10.1186/1756-0500-4-319)
Supplement: Additional file 1 — Duplication numbers of 9 most abundant CREs in top 40 highly expressed genes in sperm cells of rice. Based on the results of PLACE database SIGNALSCAN searches for total of 223 CREs, 9 of them are found to present in 1 Kb upstream regions of all 40 genes. The duplication numbers of those CREs are represented in this table. Frequency graph is also plotted for this distribution as shown in Figure 1. [file 1756-0500-4-319-S1.PDF]

Additional file1 **Duplication numbers of 9 most abundant CREs in top 40 highly expressed genes in sperm cells of rice.**

GENE ID	Duplication numbers of CREs								
	ARR1AT	CAATBOX1	CACTFTPPCA1	DOFCOREZM	GATABOX	GT1CONSENSUS	GTGANTG10	ROOTMOTIFTAPOX1	WRKY71OS
Os05g18730	10	9	13	9	10	7	9	3	5
Os09g27040	16	9	13	10	5	3	13	2	7
Os09g25650	7	12	10	11	2	8	6	3	4
Os01g42060	11	14	18	11	10	6	6	2	8
Os04g46490	7	13	12	11	3	2	11	1	10
Os03g08070	8	8	11	10	11	9	9	3	10
Os09g35720	9	11	18	11	10	14	8	4	2
Os04g29090	8	7	15	18	12	10	5	6	7
Os05g01500	7	9	20	19	6	9	11	4	7
Os03g55890	8	7	26	14	3	7	6	3	5
Os02g09580	11	14	11	9	7	4	7	11	2
Os06g20860	10	18	17	15	11	8	4	10	2
Os08g34640	8	10	24	17	6	12	3	5	5
Os03g44630	13	13	14	9	6	12	8	11	7
Os03g37570	5	6	19	4	20	7	10	7	11
Os02g19180	10	10	14	16	2	9	6	5	3
Os06g38950	11	8	10	10	19	11	5	15	5
Os11g08440	14	7	7	9	4	4	9	5	10
Os01g23580	12	12	20	9	8	5	4	8	3
Os10g02920	12	12	14	7	7	3	4	2	4
Os04g46760	15	14	13	14	9	7	4	4	8
Os03g45980	12	10	13	10	6	6	1	10	4
Os10g25060	14	7	9	10	9	6	7	4	6
Os08g16610	7	6	11	11	6	17	7	1	2
Os02g20530	13	8	21	14	7	14	13	6	8
Os02g44599	13	7	6	10	4	10	10	3	8
Os12g38460	15	6	6	11	3	10	6	2	6
Os02g08080	15	13	10	11	3	12	11	6	4
Os12g06480	10	10	18	14	13	13	9	7	10
Os02g02800	6	17	15	10	6	9	6	8	9
Os05g02030	13	8	22	4	8	7	13	7	5
Os08g35700	7	5	8	7	4	2	5	3	4
Os07g04520	13	11	11	7	5	7	4	2	7
Os05g11980	13	8	15	14	4	9	7	4	6
Os06g07130	12	11	24	18	3	5	4	10	3
Os08g28080	12	14	14	11	8	10	7	15	1
Os11g37200	11	10	17	8	11	6	10	6	4
Os05g03320	6	11	17	18	10	8	7	5	5
Os03g04690	6	14	20	12	5	5	7	7	2
Os08g05820	7	15	19	17	7	10	12	10	3
Total	417	414	595	460	293	323	294	230	222

Based on the results of PLACE database SIGNALSCAN searches for total of 223 CREs, 9 of them are found to present in 1 Kb upstream regions of all 40 genes. The duplication numbers of those CREs are represented in this table. Frequency graph is also plotted for this distribution as shown in Figure 1.
