# Supplementary material for: Putative cis-regulatory elements in genes highly expressed in rice sperm cells
Source: BMC Res Notes. 2011 Sep 5;4:319. doi: 10.1186/1756-0500-4-319 (PMC3224587; doi:10.1186/1756-0500-4-319)
Supplement: Additional file 2 — Duplication numbers of other 19 abundant CREs present in 80% of sperm cell expressing genes of rice. Results of SIGNALSCAN searches gave 19 other CREs abundantly present in the dataset. The duplication numbers of these CREs are represented in this table. Frequency graph is also plotted for this distribution as shown in Figure 2. [file 1756-0500-4-319-S2.PDF]

Additional file 2 **Duplication numbers of other 19 abundant CREs present in 80% of sperm cell expressing genes of rice.**

S. No.	CRE Name	Duplication Number
1	BIHD1OS	90
2	CCAATBOX1	87
3	CURECORECR	208
4	EBOXBNNAPA	348
5	GT1GMSCAM4	92
6	IBOXCORE	88
7	INRNTPSADB	90
8	MYBCORE	118
9	MYBST1	72
10	MYCCONSENSUSAT	348
11	NODCON2GM	96
12	OSE2ROOTNODULE	96
13	POLASIG1	97
14	POLLEN1LELAT52	189
15	RAV1AAT	104
16	SEF4MOTIFGM7S	93
17	TAAAGSTKST1	86
18	TATABOX5	97
19	WBOXNTERF3	100

Results of SIGNALSCAN searches gave 19 other CREs abundantly present in the dataset. The duplication numbers of these CREs are represented in this table. Frequency graph is also plotted for this distribution as shown in Figure 2.
