# Supplementary figures and images for: Putative cis-regulatory elements in genes highly expressed in rice sperm cells
Source: BMC Res Notes. 2011 Sep 5;4:319. doi: 10.1186/1756-0500-4-319 (PMC3224587; doi:10.1186/1756-0500-4-319)

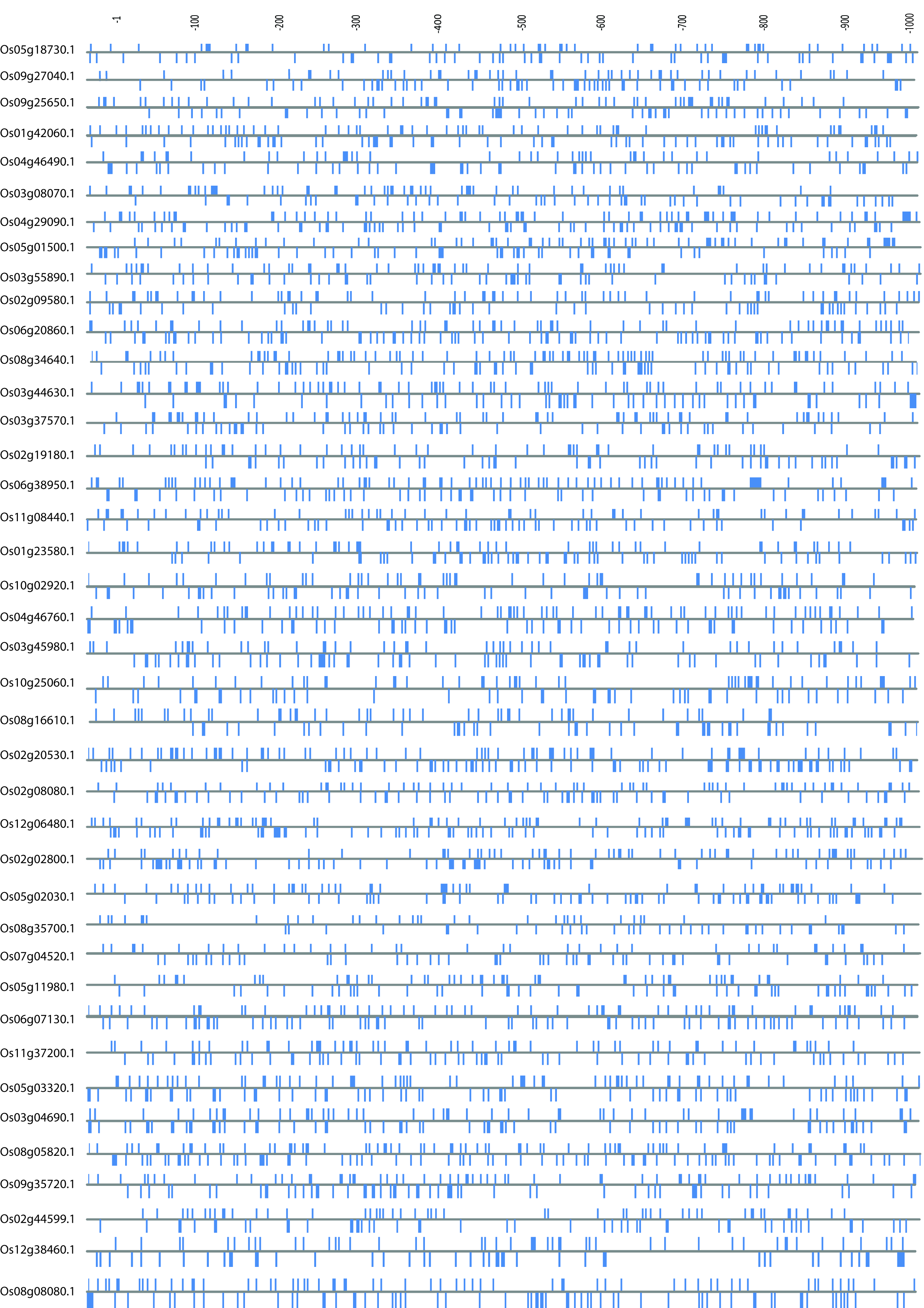

Supplement: Additional file 3 — A map of 28 abundant CREs and their positions within 1 Kb upstream sequences. SIGNALSCAN program of PLACE database identified the positions of CREs in the upstream regions of top 40 highly expressed genes in sperm cell of rice. These selected CREs were subjected to further extensive analysis for their duplication numbers and distribution across the upstream regions. The figure shows exact location of CREs present in 80% of the gene dataset. The blue bars above the horizontal black line indicate CREs on sense strand and the blue bars below the black line designates CREs on anti-sense strand. [file 1756-0500-4-319-S3.TIFF]
