# Supplementary material for: Putative cis-regulatory elements in genes highly expressed in rice sperm cells
Source: BMC Res Notes. 2011 Sep 5;4:319. doi: 10.1186/1756-0500-4-319 (PMC3224587; doi:10.1186/1756-0500-4-319)
Supplement: Additional file 4 — Unique CREs. The analysis exhibited some unique CREs present in only one of the 40 sperm cell expressing genes with one or two duplications. These CREs were found in these specific sperm cell expressing genes. [file 1756-0500-4-319-S4.PDF]

Additional file 4 **Unique CREs.**

S. No	Gene ID	CRE Name	Signal sequence	Duplication Number
1	LOC_Os10g25060.1	ABREZMRAB28	CCACGTGG	2
2	LOC_Os08g05820.1	AGCBOXNPGLB	AGCCGCC	1
3	LOC_Os08g16610.1	AMMORESIIUDCRNIA1	GGWAGGGT	1
4	LOC_Os02g19180.1	AMMORESIVDCRNIA1	CGAACTT	1
5	LOC_Os02g02800.1	ANAERO5CONSENSUS	TTCCCTGTT	1
6	LOC_Os12g06480.1	AUXREPSIAA4	KGTCCCAT	1
7	LOC_Os05g02030.1	AUXRETGA2GMGH3	TGACGTGGC	1
8	LOC_Os06g07130.1	BOX2PSGS2	TCTAAGCAAAG	1
9	LOC_Os02g19180.1	CACGCAATGMGH3	CACGCAAT	1
10	LOC_Os05g03320.1	CARGNCAT	CCWWWWWWWWGG	2
11	LOC_Os02g02800.1	CMSRE1IBSPOA	TGGACGG	1
12	LOC_Os08g05820.1	D1GMAUX28	ACAGTTACTA	1
13	LOC_Os05g18730.1	E2FBNTRNR	GCGGCAAA	1
14	LOC_Os02g44599.1	EVENINGAT	AAAATATCT	1
15	LOC_Os10g25060.1	GBOXLERBCS	MCACGTGGC	1
16	LOC_Os03g04690.1	INTRONUPPER	MAGGTAAGT	1
17	LOC_Os01g42060.1	L1BOXATPDF1	TAAATGYA	1
18	LOC_Os10g25060.1	LREBOXIIPCCHS1	TCCACGTGGC	1
19	LOC_Os10g25060.1	MARABOX1	AATAAAYAAA	1
20	LOC_Os08g05820.1	MYB26PS	GTTAGGTT	1
21	LOC_Os05g01500.1	MYBATRD22	CTAACCA	1
22	LOC_Os04g46760.1	OCTAMOTIF2	CGCGGCAT	1
23	LOC_Os05g02030.1	OPAQUE2ZMB32	GATGAYRTGG	1
24	LOC_Os03g08070.1	PALBOXPPC	YTYYMMCMAMCMMC	1
25	LOC_Os05g11980.1	PIATGAPB	GTGATCAC	2
26	LOC_Os06g20860.1	RGATAOS	CAGAAGATA	1
27	LOC_Os02g19180.1	SBOXATRBCS	CACCTCCA	1
28	LOC_Os08g05820.1	SORLIP3AT	CTCAAGTGA	1
29	LOC_Os01g23580.1	SORLREP4AT	CTCCTAATT	1
30	LOC_Os08g05820.1	SURE2STPAT21	AATACTAAT	1
31	LOC_Os08g16610.1	TATCCACHVAL21	TATCCAC	1
32	LOC_Os03g37570.1	TE2F2NTPCNA	ATTCCCGC	1
33	LOC_Os04g46490.1	TELOBOXATEEF1AA1	AAACCCTAA	1
34	LOC_Os05g02030.1	UPRE2AT	CCACGTCATC	1
35	LOC_Os12g06480.1	VSF1PVGRP18	GCTCCGTTG	1

The analysis exhibited some unique CREs present in only one of the 40 sperm cell expressing genes with one or two duplications. These CREs were found in these specific sperm cell expressing genes.
