# Supplementary material for: Putative cis-regulatory elements in genes highly expressed in rice sperm cells
Source: BMC Res Notes. 2011 Sep 5;4:319. doi: 10.1186/1756-0500-4-319 (PMC3224587; doi:10.1186/1756-0500-4-319)
Supplement: Additional file 5 — Peculiar CREs. Besides abundant CREs present in 80% of the gene dataset, there are few others present in just 5-10% of rice sperm cell expressing genes. [file 1756-0500-4-319-S5.PDF]

Additional file 5 **Peculiar CREs.**

S.No.	CRE Name	Gene ID	Signal sequence	Duplication Number
1	ABREATCONSENSUS	LOC_Os05g18730.1<br>LOC_Os10g25060.1	YACGTGGC	1<br>1
2	ABREMOTIFAOSOSEM	LOC_Os04g29090.1<br>LOC_Os03g45980.1	TACGTGTC	1<br>1
3	ACGTABREMOTIFAOSOSEM	LOC_Os04g29090.1<br>LOC_Os03g45980.1	TACGTGTC	1<br>1
4	ANAERO4CONSENSUS	LOC_Os03g55890.1<br>LOC_Os11g08440.1	GTTTHGCAA	1<br>1
5	BOXCPSAS1	LOC_Os06g20860.1<br>LOC_Os05g02030.1	CTCCCAC	1<br>1
6	CEREGLUBOX3PSLEGA	LOC_Os09g25650.1<br>LOC_Os03g08070.1	TGTAAAAGT	1<br>1
7	DRE1COREZMRAB17	LOC_Os04g46490.1<br>LOC_Os06g38950.1	ACCGAGA	1<br>1
8	EMBP1TAEM	LOC_Os05g18730.1<br>LOC_Os10g25060.1	CACGTGGC	1<br>1
9	HDZIP2ATATHB2	LOC_Os09g25650.1<br>LOC_Os09g35720.1	TAATMATTA	1<br>1
10	HEXAT	LOC_Os05g02030.1<br>LOC_Os07g04520.1	TGACGTGG	1<br>1
11	LEAFYATAG	LOC_Os02g02800.1<br>LOC_Os07g04520.1	CCAATGT	1<br>1
12	LRENPCABE	LOC_Os05g18730.1<br>LOC_Os10g25060.1	ACGTGGCA	1<br>1
13	SITEIOSPCNA	LOC_Os06g20860.1<br>LOC_Os10g25060.1	CCAGGTGG	1<br>1
14	SORLREP3AT	LOC_Os04g29090.1<br>LOC_Os03g45980.1	TGTATATAT	1<br>1
15	UPRMOTIFIAT	LOC_Os05g02030.1<br>LOC_Os07g04520.1	CCACGTCA	1<br>1
16	ZDNAFORMINGATCAB1	LOC_Os01g23580.1<br>LOC_Os11g37200.1	ATACGTGT	1<br>1
17	ACGTOSGLUB1	LOC_Os04g46490.1<br>LOC_Os04g29090.1<br>LOC_Os02g44599.1	GTACGTG	1<br>1<br>1
18	ARE1	LOC_Os05g01500.1<br>LOC_Os03g44630.1<br>LOC_Os11g37200.1	RGTGACNNNGC	1<br>1<br>1
19	BOXIIPCCHS	LOC_Os05g18730.1<br>LOC_Os10g25060.1<br>LOC_Os05g02030.1	ACGTGGC	1<br>1<br>1
20	CELLCYCLESC	LOC_Os03g08070.1<br>LOC_Os08g16610.1<br>LOC_Os12g38460.1	CACGAAAA	1<br>1<br>1
21	CEREGLUBOX2PSLEGA	LOC_Os09g25650.1<br>LOC_Os06g20860.1	TGAAAACT	1<br>1

		LOC_Os10g02920.1		1
22	CTRMCAMV35S	LOC_Os01g23580.1	TCTCTCTCT	1
		LOC_Os02g20530.1		1
		LOC_Os08g05820.1		2
23	E2F1OSPCNA	LOC_Os06g38950.1	GCGGGAAA	1
		LOC_Os08g35700.1		1
		LOC_Os05g11980.1		2
24	E2FANTRNR	LOC_Os06g38950.1	TTTCCCGC	1
		LOC_Os08g35700.1		1
		LOC_Os05g11980.1		2
25	E2FAT	LOC_Os06g38950.1	TYTCCCGCC	1
		LOC_Os08g35700.1		1
		LOC_Os05g11980.1		1
26	GCN4OSGLUB1	LOC_Os05g01500.1	TGAGTCA	1
		LOC_Os02g09580.1		1
		LOC_Os02g19180.1		1
27	IRO2OS	LOC_Os05g18730.1	CACGTGG	2
		LOC_Os10g25060.1		2
		LOC_Os08g05820.1		1
28	LTREATLTI78	LOC_Os03g55890.1	ACCGACA	1
		LOC_Os10g02920.1		1
		LOC_Os10g25060.1		1
29	MARARS	LOC_Os03g44630.1	WTTTATRTTTW	1
		LOC_Os06g07130.1		1
		LOC_Os11g37200.1		1
30	PALINDROMICCBOXGM	LOC_Os03g37570.1	TGACGTCA	2
		LOC_Os10g25060.1		2
		LOC_Os02g02800.1		2
31	QARBNEXTA	LOC_Os04g46490.1	AACGTGT	1
		LOC_Os03g08070.1		1
		LOC_Os02g20530.1		1
32	RYREPEATVFLEB4	LOC_Os03g45980.1	CATGCATG	2
		LOC_Os07g04520.1		2
		LOC_Os05g03320.1		2
33	S1FSORPL21	LOC_Os02g09580.1	ATGGTATT	1
		LOC_Os11g37200.1		1
		LOC_Os08g28080.1		1
34	TRANSINITDICOTS	LOC_Os03g44630.1	AMNAUGGC	1
		LOC_Os04g46760.1		1
		LOC_Os02g08080.1		1
35	UP1ATMSD	LOC_Os05g01500.1	GGCCCAWWW	1
		LOC_Os08g34640.1		1
		LOC_Os02g02800.1		1
36	XYLAT	LOC_Os09g25650.1	ACAAAGAA	1
		LOC_Os03g55890.1		1
		LOC_Os06g20860.1		1
37	AGMOTIFNTMYB2	LOC_Os02g19180.1	AGATCCAA	1
		LOC_Os05g03320.1		1
		LOC_Os03g04690.1		1

		LOC_Os02g44599.1		1
38	CARGATCONSENSUS	LOC_Os04g46490.1<br>LOC_Os05g01500.1<br>LOC_Os02g09580.1<br>LOC_Os06g20860.1	CCWWWWWWGG	2<br>2<br>2<br>2
39	GARE1OSREP1	LOC_Os09g25650.1<br>LOC_Os04g29090.1<br>LOC_Os03g45980.1<br>LOC_Os05g03320.1	TAACAGA	1<br>1<br>1<br>1
40	GT1MOTIFPSRBCS	LOC_Os04g46490.1<br>LOC_Os03g08070.1<br>LOC_Os02g20530.1<br>LOC_Os02g02800.1	KWGTGRWAAWRW	1<br>1<br>1<br>1
41	MYB1LEPR	LOC_Os04g29090.1<br>LOC_Os05g01500.1<br>LOC_Os05g02030.1<br>LOC_Os07g04520.1	GTTAGTT	1<br>2<br>1<br>1
42	PROXBBNNAPA	LOC_Os05g01500.1<br>LOC_Os02g09580.1<br>LOC_Os08g35700.1<br>LOC_Os09g35720.1	CAAACACC	1<br>1<br>1<br>1
43	QELEMENTZMZM13	LOC_Os03g55890.1<br>LOC_Os03g37570.1<br>LOC_Os11g08440.1<br>LOC_Os07g04520.1	AGGTCA	1<br>1<br>1<br>2
44	UP2ATMSD	LOC_Os04g46490.1<br>LOC_Os05g11980.1<br>LOC_Os11g37200.1<br>LOC_Os12g38460.1	AAACCCTA	1<br>1<br>1<br>1

Besides abundant CREs present in 80% of the gene dataset, there are few others present in just 5-10% of rice sperm cell expressing genes.
